# Novel Epigenetic Changes Unveiled by Monozygotic Twins Discordant for Smoking Habits

**DOI:** 10.1371/journal.pone.0128265

**Published:** 2015-06-04

**Authors:** Alessandra Allione, Francesca Marcon, Giovanni Fiorito, Simonetta Guarrera, Ester Siniscalchi, Andrea Zijno, Riccardo Crebelli, Giuseppe Matullo

**Affiliations:** 1 Human Genetics Foundation, HuGeF, Turin, Italy; 2 Department of Medical Sciences, University of Turin, Turin, Italy; 3 Department of Environment and Primary Prevention, Istituto Superiore di Sanità, Rome, Italy; UCLA-DOE Institute for Genomics and Proteomics, UNITED STATES

## Abstract

Exposure to cigarette smoking affects the epigenome and could increase the risk of developing diseases such as cancer and cardiovascular disorders. Changes in DNA methylation associated with smoking may help to identify molecular pathways that contribute to disease etiology. Previous studies are not completely concordant in the identification of differentially methylated regions in the DNA of smokers. We performed an epigenome-wide DNA methylation study in a group of monozygotic (MZ) twins discordant for smoking habits to determine the effect of smoking on DNA methylation. As MZ twins are considered genetically identical, this model allowed us to identify smoking-related DNA methylation changes independent from genetic components. We investigated the whole blood genome-wide DNA methylation profiles in 20 MZ twin pairs discordant for smoking habits by using the Illumina HumanMethylation450 BeadChip. We identified 22 CpG sites that were differentially methylated between smoker and non-smoker MZ twins by intra-pair analysis. We confirmed eight loci already described by other groups, located in *AHRR*, *F2RL3*, *MYOG1* genes, at 2q37.1 and 6p21.33 regions, and also identified several new loci. Moreover, pathway analysis showed an enrichment of genes involved in GTPase regulatory activity. Our study confirmed the evidence of smoking-related DNA methylation changes, emphasizing that well-designed MZ twin models can aid the discovery of novel DNA methylation signals, even in a limited sample population.

## Introduction

The term “epigenetics” refers to heritable changes in gene function, which do not involve changes to the underlying DNA sequence; a change in phenotype without a change in genotype. Epigenetic changes arising during a lifetime are likely to be triggered by individual endogenous factors as well as by environmental factors. Given the central role played by the progressive accumulation of epigenetic changes in the etiopathology of cancer and other degenerative diseases [1], there is growing interest in the effect of modification on disease risk exerted by environmental and lifestyle factors, which may ultimately alter the risk of developing cancer. Among lifestyle and behavioral factors, smoking habits deserve major consideration. In fact, epigenetic alterations are likely to play a role in tobacco-induced cancers; an association between smoking and altered DNA methylation of specific genes in lung cancer tissue has been shown [2], as well as the progressive accumulation of epigenetic alterations in the respiratory epithelium of heavy smokers during the carcinogenic process [3, 4]. In addition, recent epigenome-wide association studies identified several genetic loci showing smoking-related DNA methylation changes in DNA from blood [3, 5–10], supporting the use of this surrogate non-tumor tissue to detect altered DNA methylation in smokers.

Monozygotic (MZ) twins provide a powerful model to assess the influence of environmental factors on a particular trait of interest; in subjects who are genetically identical but experience different exposure to environmental or lifestyle factors, any differences can be directly ascribed to this non-shared environment experienced by co-twins [11, 12]. Thus, genetically identical individuals can provide a convenient model to evaluate epigenetic changes where there is no genetic variation.

Taking advantage of the MZ twin-based design, we investigated the effects of smoke exposure on genomic DNA methylation in a population of co-twins with discordant smoking habits [13].

The use of MZ twin pairs who were discordant for smoking habits allowed a perfect match both for the genetic component and the *in utero* and early childhood environmental exposures between smoker and non-smoker co-twins, thus greatly increasing the sensitivity of the analysis. In the present investigation we tested, on a genome-wide level, whether smoking duration and/or intensity could modulate gene-specific methylation in blood DNA.

## Material and Methods

### Sample description

A total of 21 MZ twin pairs who were discordant for smoking habits were enrolled in this cross-sectional study, which was conducted in accordance with the principles of Good Clinical Practice and approved by the Independent Ethics Committee of the University of Rome ‘‘Tor Vergata”. Written informed consent was obtained from all subjects according to the Declaration of Helsinki. Information on demographics, medical history, lifestyle, dietary habits, occupational and environmental exposures was collected by means of a questionnaire. Saliva samples were taken from all subjects to verify the zygosity status. Testing for zygosity was performed using the AmpFiSTRs Identifier kit (Applied Biosystems) [13]. All analyses were carried out on coded samples.

### Smoking history

Detailed information on smoking history was collected for all subjects as previously described [13, 14]. Smoking status was assigned as follows: a) smokers: each member of the MZ twin pair that smoked; all smokers had a tar intake of ≥ 60 mg tar/day, based on the number of cigarettes smoked per day multiplied by the tar yield of the cigarette brand as declared on the pack (this cut-off was chosen to exclude irregular or occasional smokers); b) non-smokers: each member of the MZ twin pair who has never smoked or had stopped smoking since more than 10 years. To avoid misclassifications based on the self-reported smoker or non-smoker status, urinary cotinine levels were measured in all study subjects using the Accutest NicAlert_test kit (Jant Pharmacal Corp.) according to the manufacturer’s instructions. Overall tobacco exposure during tha smoker’s lifetime was quantified by the pack-year parameter which integrates the number of cigarettes smoked per day during the smoking period. In particular, one pack-year is defined as 20 manufactured cigarettes (one pack) smoked per day for one year, and the number of pack-years for each smoker was calculated as (number of cigarettes smoked per day × number of years smoked)/20.

### Blood sample collection

Blood samples from all donors were collected within 2 days. Members of the same twin-pair were sampled on the same day, and blood samples were processed in parallel. Differential white blood cells (WBCs) count was determined for each sample, as previously described [13].

### DNA methylation array

Genomic DNA was isolated from whole blood samples using Puregene Core Kit (Qiagen, Hilden, Germany), according to the manufacturer’s instructions and stored at −80°C. Bisulphite conversion of 500 ng of each sample was performed using the EZ-96 DNA Methylation Gold kit (Zymo Research, Orange, CA, USA). Bisulphite-converted DNA was hybridized on the Illumina HumanMethylation450 BeadChip (Illumina Inc., San Diego, CA), following the Illumina Infinium HD methylation protocol.

### Statistical analyses

The GenomeStudio Methylation Module v1.0 software (Illumina) was used to convert on-chip fluorescent methylation signals into numerical values (β-values) between 0 and 1, which represent the methylation percentage of each analyzed CpG site.

Raw methylation data were normalized and analyzed according to standard procedures using the open source R statistical environment Bioconductor (methylumi package) [15]. The following criteria were used to filter out samples, β-values, and CpG sites respectively: global call rate ≤ 95%, detection p-value ≥ 0.01, and detection p-value ≥ 0.01 in more than 20% of the assayed samples. To identify differentially methylated CpGs between smokers and non-smokers, we applied the paired Kruskal-Wallis non parametric test. CpG loci with p<0.001, and mean methylation differences of at least 0.03 and greater than 3 standard deviations from the mean were selected and further investigated. In order to ascertain whether inter-individual variability in whole blood cell composition could affect the results, for each of the identified differentially methylated sites we compared the estimated differences in methylation by two multivariate linear regression models: the first including cell type composition (from each subject’s WBC count) as covariate, and the second without adjusting for cell type.

To investigate the association between time and smoking intensity with DNA-methylation levels of the CpG sites identified in the previous analysis, Spearman correlation coefficients between intra-pair differences (non-smokers vs smokers) in methylation and pack-years were calculated.

### Enrichment analysis

Enrichment analysis was carried out using the Gene Ontology (GO) tool “WEB-based GEne SeT AnaLysis Toolkit” (WebGestalt; http://bioinfo.vanderbilt.edu/webgestalt/; [16, 17]). A list of 223 genes in which the top differentially methylated CpGs between smokers and non-smokers were located (p<0.001; S1 Table) was uploaded, 216 of which were recognized by the software and analyzed.

## Results

The final dataset included 20 twin pairs. A total of 436,184 CpG sites passed the quality control, 22 of which (Table 1; CpG list ordered by Mean ∆β) had differential methylation levels associated with smoking status (selected as described in the Materials and Methods section). No significant difference was observed comparing DNA methylation between smokers and non-smokers with and without adjusting for WBC proportions, thus we described the results obtained without any adjustment for WBC count.

**Table 1:** DNA methylation differences at specific loci between smoking and non-smoking discordant twin pairs.

ProbeID	Chr	Position	Gene	Mean ∆β	SD	p-value	Previously reported
cg05575921	5	373378	*AHRR*	-0.140	0.018	1.91x10^-6^	Besingi and Johansson 2014; Breitling et al. 2011; Dogan et al. 2014; Elliott et al. 2014; Harlid et al. 2014; Monick et al. 2012; Shenker et al. 2013; Zeilinger et al. 2013
cg21566642	2	233284661		-0.088	0.015	4.77x10^-5^	Monick et al. 2012 (lung macrophages); Shenker et al. 2013; Zeilinger et al. 2013
cg05951221	2	233284402		-0.073	0.013	5.72x10^-6^	Monick et al. 2012 (lung macrophages); Harlid et al. 2014; Shenker et al. 2013
cg06126421	6	30720080		-0.062	0.010	2.10x10^-4^	Besingi and Johansson 2014; Dogan et al. 2014; Elliott et al. 2014; Shenker et al. 2013; Zeilinger et al. 2013
cg01940273	2	233284934		-0.060	0.011	5.72x10^-6^	Shenker et al. 2013; Zeilinger et al. 2013
cg01961092	4	84373320	*HELQ*	-0.047	0.006	4.83x10^-4^	
cg03636183	19	17000585	*F2RL3*	-0.046	0.009	1.91x10^-5^	Besingi and Johansson 2014; Harlid et al. 2014; Shenker et al. 2013; Wan et al. 2012; Zeilinger et al. 2013
cg25648203	5	395444	*AHRR*	-0.042	0.007	8.20x10^-5^	Breitling et al. 2011; Shenker et al. 2013; Zeilinger et al. 2013; Monick et al. 2012 (lung macrophages)
cg13437870	6	52909126	*ICK*	-0.040	0.007	4.83x10^-4^	
cg04074536	4	798711	*CPLX1*	-0.038	0.009	5.86x10^-4^	
cg03650233	8	57360226	*PENK*	-0.038	0.007	7.08x10^-4^	
cg12224879	2	46973883	*SOCS5*	-0.037	0.005	2.61x10^-4^	
cg09022230	7	5457225	*TNRC18*	-0.037	0.008	3.22x10^-4^	
cg10655371	7	91749682	*CYP51A1*	-0.036	0.007	5.86x10^-4^	
cg26979044	16	25045552		-0.036	0.006	1.68x10^-4^	
cg02339888	1	67862336	*IL12RB2*	-0.034	0.006	4.83x10^-4^	
cg08681409	10	134697042		-0.032	0.007	2.10x10^-4^	
cg23261204	10	133733416		-0.032	0.004	4.83x10^-4^	
cg02567879	1	11761416	*C1orf187*	-0.030	0.007	3.95x10^-4^	
cg01157780	20	34700376	*EPB41L1*	0.031	0.006	1.05x10^-4^	
cg06935361	13	32964454	*BRCA2*	0.049	0.006	7.08x10^-4^	
cg12803068	7	45002919	*MYO1G*	0.083	0.013	3.95x10^-4^	Zeilinger et al. 2013

Out of the 22 CpGs with differential methylation levels, 15 were located in known genes. The majority of these CpGs (86%) were found to be less methylated in smokers. The most significant CpG site, cg05575921 (Δ smokers vs non-smokers -14%, p = 1.91x10^-6^), was located in the *AHRR* gene. The second region was on chromosome 2q37.1 and comprised three differentially methylated CpG sites (Δ smokers vs non-smokers: cg21566642–8.8%, p = 4.77x10^-5^; cg233284402–7.3%, p = 5.72x10^-6^; cg233284934–6%, p = 5.72x10^-6^, respectively).

The intergenic region at 6p21.33 was located in a gene desert region and included the CpG locus cg06126421, which is less methylated in smokers than in non-smokers (Δ smokers vs non-smokers = -6.2%, p = 2.10x10^-4^).

We found DNA hypomethylation in smokers in the cg01961092 site, located in the *HELQ* gene body (Δ smokers vs non-smokers = -4.7%, p = 4.83x10^-4^) and we also report a significant association of tobacco smoking with the CpG site cg03636183, located within the *F2RL3* gene (Δ smokers vs non-smokers = -4.6%, p = 1.91x10^-5^).

Other hypomethylated sites in smokers with a difference > 3% were located in the following genes: *ICK*, *CPLX1*, *PENK*, *SOCS5*, *TNRC18*, *CYP51A1* and *IL12RB2* or in regions with no annotated transcripts.Three sites resulted as being hypermethylated in smokers, which were located in the *MYO1G* (cg12803068), *BRCA2* (cg06935361) and *EPB41L1* (cg01157780) genes.

**Fig 1:**
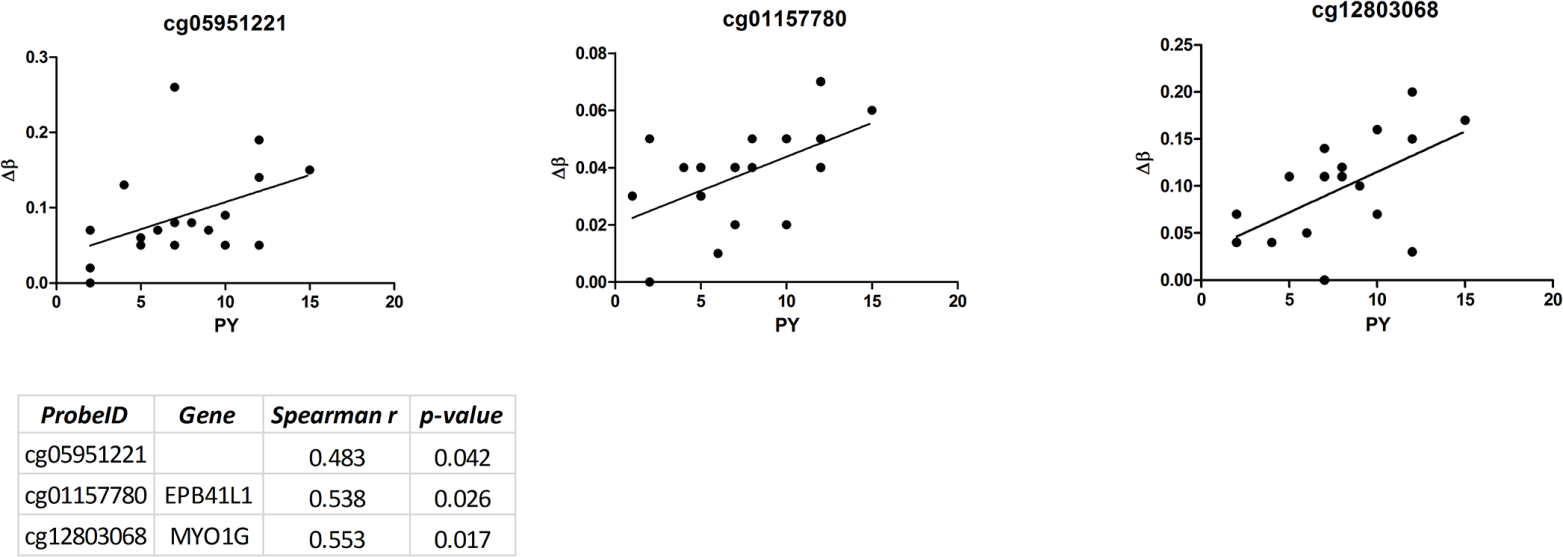
Correlation between methylation levels and smoking intensity (expressed as pack-years) for 2q37.1, *EPB41L1* and *MYO1G* loci.

Moreover, a positive correlation between methylation Δs (as described in the Materials and Methods section) and smoking intensity (expressed as pack-years) was found for three of the above mentioned loci: 2q37.1, *EPB41L1* and *MYO1G* (Fig 1).

For the gene-set enrichment analysis, a list of 223 genes in which CpGs differentially methylated between smokers and non-smokers were found to be located (S1 Table) was considered, 216 of which were recognized by WebGestalt software and analyzed for GO enrichment analysis. A total of six molecular function categories (Table 2) were significantly enriched (FDR q-value >0.05). The most enriched molecular function categories were all related to GTPase regulatory activity; in particular the Rho- and Ras-guanyl-nucleotide exchange factor activity categories were the most enriched (FDR q-value = 0.0158 for both). The genes harboring differentially methylated CpGs that were classified as members of the “small GTPase regulator activity”, “Rho guanyl-nucleotide exchange factor activity” and “Ras guanyl-nucleotide exchange factor activity” categories were: *PSD4*, *TBC1D22A*, *MCF2L*, *RASGRF2*, *IQSEC1*, *KALRN*, *OBSCN*, *DENND3*, *ARHGEF16*.

**Table 2:** Gene enrichment results carried out using the Gene Ontology (GO) enrichment analysis web-based bioinformatics tool WEB-based GEne SeT AnaLysis Toolkit (WebGestalt).

**Molecular functions that were enriched for genes differentially methylated in smokers**				
**GO Term**	**Description**	**p-value**	**FDR q-value**	**Genes**
GO:0005083	small GTPase regulator activity	0.0004	0.0158	*PSD4*, *TBC1D22A*, *MCF2L*, *RASGRF2*, *IQSEC1*, *KALRN*, *OBSCN*, *DENND3*, *ARHGEF16*
GO:0005085	guanyl-nucleotide exchange factor activity	9.85x10^-05^	0.0158	*PSD4*, *MCF2L*, *RASGRF2*, *IQSEC1*, *KALRN*, *OBSCN*, *DENND3*, *ARHGEF16*
GO:0005089	Rho guanyl-nucleotide exchange factor activity	0.0005	0.0158	*MCF2L*, *RASGRF2*, *KALRN*, *OBSCN*, *ARHGEF16*
GO:0005088	Ras guanyl-nucleotide exchange factor activity	0.0003	0.0158	*MCF2L*, *RASGRF2*, *KALRN*, *OBSCN*, *DENND3*, *ARHGEF16*
GO:0005543	phospholipid binding	0.0005	0.0158	*IRS2*, *DOC2A*, *SH3BP2*, *PSD4*, *MCF2L*, *RASGRF2*, *OSBPL5*, *IQSEC1*, *OBSCN*, *KALRN*, *APOA1*, *ARHGEF16*
GO:0070644	vitamin D response element binding	0.0004	0.0158	*TCF3*, *RXRA*
**Cellular components that were enriched for genes differentially methylated in smokers**				
**GO Term**	**Description**	**p-value**	**FDR q-value**	**Genes**
GO:0017053	transcriptional repressor complex	0.0003	0.0483	*TFAP4*, *NCOR2*, *ZFPM1*, *PRDM16*, *MTA1*

## Discussion

In order to investigate gene-specific DNA methylation changes associated with smoking habits we used a high-throughput whole-genome approach in a sample of MZ twins discordant for smoking habits. Comparing discordant MZ twins represents a powerful improvement over the traditional case-control study to search for disease-associated biological markers and environmental exposure-related phenotypes. The strength of this design to unveil epigenetic effects was demonstrated in recent studies which compared the DNA methylation patterns of MZ twins who were discordant for various diseases [18–22]. Direct comparisons between identical twins constitute an ideal experimental model for testing environmental epigenetics because differences in DNA sequences, including single-nucleotide polymorphisms (SNPs) abundant in singleton-based studies, which could potentially modulate the effects of environmental exposures or the DNA methylation levels, do not interfere in the analysis.

Several of the most significant smoking-associated hypomethylated CpG sites that we have described here have been already reported in the literature (see Table 1).

The underlying relationship of AHRR hypomethylation and smoking is further supported by Monick *et al*. [23], who reported a significant association between cg05575921 DNA methylation and AHRR gene expression and by Philibert *et al*. [24], showing a high correlation of *AHRR* DNA methylation and serum cotinine levels in smokers. The latter concluded that *AHRR* DNA methylation status is a sensitive marker of smoking history, which could help with integrating self-reported smoking habits or existing smoking-related biomarkers used in clinical or epidemiological settings (e.g., cotinine). In addition, if properly configured as a clinical assay, *AHRR* methylation levels could also be used as a screening tool in efforts to target antismoking interventions to nascent smokers in the early phases of smoking.

We found a novel CpG hypomethylated in smokers (cg01961092) located in the *HELQ* gene body. HELQ is a 3’–5’ DNA helicase with strand displacement activity that was recently shown to participate in resistance to the DNA interstrand crosslinking pathway [25], caused also by aldehydes such as acrolein and crotonaldehyde, found in tobacco smoke [26].

The *F2RL3* (coagulation factor II receptor-like 3) gene encodes for a protease-activated receptor-4 (PAR-4), which has been suggested to be involved in the pathophysiology of both cardiovascular and neoplastic diseases. These patterns suggest that the adverse health effects of smoking could be partly mediated by pathways through *F2RL3* methylation. Recently Zhang *et al*., showed that *F2RL3* methylation intensity is strongly correlated with smoking status [27] and is a predictor of smoking-related mortality [28].

No clear biological relationships between smoking and other identified genes (*CPLX1*, *PENK*, *SOCS5*, *IL12RB2*, *CYP51A1*, *TNRC18*, *ICK*) are evident, however some of these genes have been previously reported as smoking-associated.

We also found CpG loci with higher DNA methylation levels in smokers located in the *MYO1G*, *BRCA2* and *EPB41L1* genes (Table 1).

The *MYO1G* cg12803068 site and other CpGs in the same gene have been already described as hypermethylated in smokers in previous studies [7, 9, 29]. MYO1G is a plasma membrane-associated class I myosin that is abundant in T and B lymphocytes and mast cells [30], and contributes to cell elasticity [31]. Altered expression of *MYO1G* could contribute in explaining the increased fibrosis found in several smoking-targeted tissues.

None of the reported differentially methylated loci reached a Bonferroni-corrected p-value threshold (p = 1.15x10^-7^). However, this statistical correction is likely to be too conservative given the non-independence of CpG sites and the identical MZ twin intra-pair genetic background. In view of the above considerations, in order to identify differentially methylated loci, we used a combined analytical approach which took into account both the statistical significance (with p<0.001 as an arbitrary threshold) and the extent of methylation changes (SD and mean β-value differences). This analytical approach is widely used in genome-wide gene expression studies, and is reported to identify loci with high reproducibility and biological relevance compared with conventional methods that merely rely on single CpG statistical significance. Moreover, the usefulness of this approach is supported by the identification (also in our study) of differentially methylated loci already described in association with smoking, showing the same methylation trends as those previously reported.

MZ twins provide a valuable model to identify environmental effects without the influence of genetic variability. Nevertheless, given the relatively small sample size, replication of our newly identified results in larger samples is needed.

The difference we observed in *AHRR* mean methylation levels (14%, range 1–32%) between smokers and non-smokers was comparable with those reported in larger non-twin independent populations studied by other authors (17% in [3]; 24.4% in [29]; 7.4% in [9]; 15% in [8]; 22% in [7]). However, large-scale epigenetic studies are still ongoing, and a threshold to define the minimum DNA methylation changes able to exert functional significance at the population level has not yet been firmly established.

To examine the possible functional impact of differential methylation at loci reported there, we conducted an exploratory analysis using a gene expression/methylation dataset produced by our group in the context of a previous study [32].

The database comprises paired gene expression and methylation data (whole blood) from the same subjects and allows methylation/gene expression correlation analyses. Unfortunately, most of the genes we identified as differentially methylated in this study were only weakly or not expressed, and only the *HELQ* gene showed evidence of association between gene expression and DNA methylation (Spearman correlation, cg01961092 methylation/gene expression r = -0.26, p = 0.02). Recently, a large-scale genome-wide expression profiling has been performed, but none of our differently methylated genes was found to be differentially expressed in smokers’ blood cells [33].

We also investigated the relationship between methylation levels and smoking intensity expressed as pack-year. We observed that in three loci, 2q37.1, *EPB41L1*, and *MYO1G*, the methylation differences between smokers and non-smokers increased according to pack-year, indicating that these three loci could be further used as epigenetic markers of smoking intensity.

The most enriched GO molecular functions were all related to GTPase regulatory activity. Interestingly, the same pathways were identified by analyzing differentially methylated genes in alveolar macrophages from smokers [23]. Moreover, molecular functions of these genes are of special interest since a recent study described gene-smoking interactions, identifying several single nucleotide polymorphisms located in genes belonging to the Rho-GTPase pathway associated with an early-onset of coronary artery disease in smokers [34]. Another enriched molecular function was “vitamin D response element binding”. Serum vitamin D levels are inversely correlated with the incidence, prevalence and death rates of different tumors [35], and recently Zheng *et al*. suggested a protective effect of vitamin D against colorectal cancer, whose effects are more beneficial in active smokers than in non-smokers [36]. Moreover, vitamin D deficiency have been associated with a diminished lung function and more rapid lung function decline in smokers in a longitudinal cohort of elderly men [37]. These results suggest that vitamin D may have a protective effect against the damaging effects of smoking on lung function. Smoking has a significant effect on calcium and vitamin D metabolism: the repression of the vitamin D-parathyroid hormone system, evident among smokers may represent another potential mechanism for the deleterious effects of smoking on bone metabolism, and may contribute to the reported risk of osteoporosis among smokers [38].

## Conclusions

In this study, we successfully identified 22 DNA methylation sites that were differentially methylated. By using MZ twins who were discordant for smoking habits, we were able to replicate most of the previously published significant sites, with a relatively small number of phenotypically discordant twin pairs. Moreover, we were also able to detect novel smoking-related differentially methylated CpG sites. Considering both our results along with previously published results, it is becoming evident that environmental exposures, such as smoking, may modify the epigenetic profiles of several genes. Given the major burden to individuals and to society of smoking-related diseases, further functional studies are warranted to better explain the pathogenic role exerted by smoking through epigenetic changes.

## Supporting Information

S1 Table List of genes where the top differentially methylated CpGs between smokers and non-smokers were located (p<0.0001). (DOCX)
