# Supplementary material for: Novel Epigenetic Changes Unveiled by Monozygotic Twins Discordant for Smoking Habits
Source: PLoS One. 2015 Jun 4;10(6):e0128265. doi: 10.1371/journal.pone.0128265 (PMC4456379; doi:10.1371/journal.pone.0128265)
Supplement: S1 Table — (DOCX) [file pone.0128265.s001.docx]

**S1 Table.** List of genes where the top differentially methylated CpGs between smokers and non-smokers were located (p<0.0001).

|  |  |  |  | ***Average β values*** | |  |  |  |
| --- | --- | --- | --- | --- | --- | --- | --- | --- |
| ***ProbeID*** | ***Chr*** | ***Position*** | ***Gene*** | ***nonsmokers*** | ***smokers*** | ***Mean ∆β*** | ***p-value*** |  |
| cg05575921 | 5 | 373378 | AHRR | 0.849 | 0.709 | -0.140 | 1.91E-06 |  |
| cg21566642 | 2 | 233284661 |  | 0.562 | 0.474 | -0.088 | 4.77E-05 |  |
| cg05951221 | 2 | 233284402 |  | 0.502 | 0.429 | -0.073 | 5.72E-06 |  |
| cg06126421 | 6 | 30720080 |  | 0.845 | 0.782 | -0.062 | 2.10E-04 |  |
| cg01940273 | 2 | 233284934 |  | 0.679 | 0.620 | -0.060 | 5.72E-06 |  |
| cg01961092 | 4 | 84373320 | HELQ | 0.826 | 0.779 | -0.047 | 4.83E-04 |  |
| cg03636183 | 19 | 17000585 | F2RL3 | 0.724 | 0.679 | -0.046 | 1.91E-05 |  |
| cg25648203 | 5 | 395444 | AHRR | 0.852 | 0.811 | -0.042 | 8.20E-05 |  |
| cg13437870 | 6 | 52909126 | ICK | 0.794 | 0.755 | -0.040 | 4.83E-04 |  |
| cg04074536 | 4 | 798711 | CPLX1 | 0.829 | 0.791 | -0.038 | 5.86E-04 |  |
| cg03650233 | 8 | 57360226 | PENK | 0.868 | 0.831 | -0.038 | 7.08E-04 |  |
| cg12224879 | 2 | 46973883 | SOCS5 | 0.723 | 0.686 | -0.037 | 2.61E-04 |  |
| cg02349373 | 19 | 38281559 |  | 0.659 | 0.623 | -0.037 | 7.08E-04 |  |
| cg09022230 | 7 | 5457225 | TNRC18 | 0.753 | 0.717 | -0.037 | 3.22E-04 |  |
| cg10655371 | 7 | 91749682 | CYP51A1 | 0.770 | 0.734 | -0.036 | 5.86E-04 |  |
| cg26979044 | 16 | 25045552 |  | 0.721 | 0.686 | -0.036 | 1.68E-04 |  |
| cg02339888 | 1 | 67862336 | IL12RB2 | 0.621 | 0.587 | -0.034 | 4.83E-04 |  |
| cg08681409 | 10 | 134697042 |  | 0.756 | 0.724 | -0.032 | 2.10E-04 |  |
| cg23261204 | 10 | 133733416 |  | 0.759 | 0.727 | -0.032 | 4.83E-04 |  |
| cg02567879 | 1 | 11761416 | C1orf187 | 0.687 | 0.657 | -0.030 | 3.95E-04 |  |
| cg13871826 | 1 | 86045347 | CYR61;DDAH1 | 0.822 | 0.792 | -0.030 | 9.54E-06 |  |
| cg16266126 | 1 | 153957455 | RAB13 | 0.830 | 0.801 | -0.029 | 8.51E-04 |  |
| cg01164584 | 8 | 67454340 |  | 0.871 | 0.843 | -0.028 | 8.51E-04 |  |
| cg04343883 | 1 | 202162338 | LGR6 | 0.786 | 0.757 | -0.028 | 4.83E-04 |  |
| cg11853283 | 5 | 824299 | ZDHHC11 | 0.837 | 0.808 | -0.028 | 4.83E-04 |  |
| cg26361535 | 8 | 144576604 | ZC3H3 | 0.754 | 0.726 | -0.028 | 2.61E-04 |  |
| cg07904567 | 12 | 133346306 | GOLGA3 | 0.853 | 0.826 | -0.028 | 8.51E-04 |  |
| cg10109731 | 16 | 88472541 |  | 0.784 | 0.756 | -0.028 | 2.61E-04 |  |
| cg08877188 | 3 | 195943579 | OSTalpha | 0.781 | 0.753 | -0.028 | 1.05E-04 |  |
| cg11948339 | 1 | 40044951 |  | 0.847 | 0.820 | -0.028 | 5.86E-04 |  |
| cg07740640 | 7 | 97841941 | BHLHA15 | 0.886 | 0.859 | -0.027 | 5.86E-04 |  |
| cg24378940 | 4 | 7742291 | SORCS2 | 0.878 | 0.851 | -0.026 | 8.51E-04 |  |
| cg11560197 | 5 | 140182956 | PCDHA2;PCDHA1;PCDHA3 | 0.460 | 0.434 | -0.026 | 5.86E-04 |  |
| cg04064050 | 6 | 127663586 | ECHDC1 | 0.875 | 0.850 | -0.025 | 5.86E-04 |  |
| cg24867215 | 19 | 18709521 | CRLF1 | 0.830 | 0.806 | -0.024 | 2.61E-04 |  |
| cg17270497 | 7 | 73256360 | WBSCR27 | 0.870 | 0.846 | -0.024 | 7.08E-04 |  |
| cg01895439 | 14 | 102974240 | ANKRD9 | 0.801 | 0.778 | -0.024 | 4.83E-04 |  |
| cg02660541 | 16 | 3193410 |  | 0.877 | 0.853 | -0.024 | 3.22E-04 |  |
| cg01972688 | 11 | 45928848 | C11orf94 | 0.800 | 0.777 | -0.023 | 1.68E-04 |  |
| cg03775330 | 19 | 4682727 | DPP9 | 0.891 | 0.868 | -0.023 | 1.68E-04 |  |
| cg00570469 | 19 | 36602113 |  | 0.914 | 0.892 | -0.022 | 5.86E-04 |  |
| cg25107528 | 4 | 8607947 | CPZ | 0.857 | 0.836 | -0.022 | 3.95E-04 |  |
| cg26306091 | 2 | 241076551 | MYEOV2 | 0.796 | 0.775 | -0.021 | 3.22E-04 |  |
| cg06672250 | 8 | 21914593 | EPB49 | 0.707 | 0.686 | -0.021 | 8.51E-04 |  |
| cg02048657 | 5 | 1293231 | TERT | 0.810 | 0.789 | -0.021 | 4.83E-04 |  |
| cg23314514 | 14 | 104852775 |  | 0.843 | 0.823 | -0.021 | 2.67E-05 |  |
| cg14479198 | 5 | 80255793 | RASGRF2 | 0.688 | 0.667 | -0.021 | 4.83E-04 |  |
| cg01076051 | 5 | 130329564 |  | 0.887 | 0.867 | -0.021 | 7.08E-04 |  |
| cg08709672 | 1 | 206224334 | AVPR1B | 0.618 | 0.598 | -0.020 | 6.29E-05 |  |
| cg03962783 | 11 | 72312062 | PDE2A | 0.927 | 0.907 | -0.020 | 7.08E-04 |  |
| cg12732638 | 8 | 142455333 | FLJ43860 | 0.820 | 0.800 | -0.020 | 3.95E-04 |  |
| cg05389935 | 1 | 26610757 | UBXN11 | 0.852 | 0.832 | -0.020 | 4.83E-04 |  |
| cg07449499 | 6 | 939195 |  | 0.810 | 0.791 | -0.019 | 3.62E-05 |  |
| cg10254082 | 7 | 997346 |  | 0.847 | 0.828 | -0.019 | 4.83E-04 |  |
| cg03443762 | 17 | 25676827 |  | 0.872 | 0.853 | -0.019 | 2.67E-05 |  |
| cg02383285 | 6 | 169286348 |  | 0.907 | 0.888 | -0.019 | 2.61E-04 |  |
| cg25810857 | 17 | 75279120 | SEPT9 | 0.952 | 0.933 | -0.019 | 1.91E-05 |  |
| cg08591998 | 7 | 73466006 | ELN | 0.924 | 0.906 | -0.018 | 7.08E-04 |  |
| cg17931320 | 19 | 885759 | MED16 | 0.925 | 0.907 | -0.018 | 2.67E-05 |  |
| cg22562363 | 10 | 123748615 | TACC2 | 0.837 | 0.819 | -0.018 | 2.10E-04 |  |
| cg03630015 | 2 | 219747459 | WNT10A | 0.783 | 0.765 | -0.018 | 8.51E-04 |  |
| cg16416158 | 1 | 21652298 | ECE1 | 0.700 | 0.682 | -0.018 | 2.10E-04 |  |
| cg01236384 | 11 | 1247831 | MUC5B | 0.865 | 0.848 | -0.018 | 4.77E-05 |  |
| cg07249888 | 15 | 101847305 | PCSK6 | 0.870 | 0.852 | -0.018 | 7.08E-04 |  |
| cg04415736 | 14 | 100798844 |  | 0.915 | 0.897 | -0.017 | 8.51E-04 |  |
| cg01037703 | 22 | 45014547 |  | 0.898 | 0.881 | -0.017 | 2.61E-04 |  |
| cg20083995 | 8 | 101969612 |  | 0.870 | 0.853 | -0.017 | 3.22E-04 |  |
| cg26469982 | 17 | 78923932 | RPTOR | 0.902 | 0.884 | -0.017 | 3.95E-04 |  |
| cg03787711 | 7 | 2756395 |  | 0.864 | 0.847 | -0.017 | 3.95E-04 |  |
| cg05844798 | 20 | 3062344 |  | 0.844 | 0.827 | -0.017 | 4.83E-04 |  |
| cg06268956 | 11 | 2423196 | TSSC4 | 0.827 | 0.810 | -0.017 | 7.08E-04 |  |
| cg15872043 | 3 | 183960177 | ALG3 | 0.905 | 0.889 | -0.017 | 8.20E-05 |  |
| cg25977879 | 12 | 133085193 | FBRSL1 | 0.938 | 0.921 | -0.017 | 8.20E-05 |  |
| cg23195199 | 4 | 89723908 | FAM13A | 0.903 | 0.886 | -0.016 | 5.86E-04 |  |
| cg01843768 | 7 | 2078650 | MAD1L1 | 0.826 | 0.810 | -0.016 | 4.83E-04 |  |
| cg06617202 | 1 | 205038787 | CNTN2 | 0.847 | 0.831 | -0.016 | 3.95E-04 |  |
| cg17787988 | 2 | 113954732 | PSD4;LOC440839 | 0.916 | 0.900 | -0.015 | 7.08E-04 |  |
| cg05330593 | 16 | 30768675 | PHKG2 | 0.919 | 0.904 | -0.015 | 3.22E-04 |  |
| cg11800390 | 7 | 5515995 | FBXL18 | 0.918 | 0.902 | -0.015 | 1.05E-04 |  |
| cg03027739 | 7 | 127805680 |  | 0.688 | 0.673 | -0.015 | 2.61E-04 |  |
| cg10604040 | 11 | 65559109 | OVOL1 | 0.841 | 0.825 | -0.015 | 5.86E-04 |  |
| cg17608570 | 10 | 130228545 |  | 0.584 | 0.569 | -0.015 | 2.61E-04 |  |
| cg22691028 | 12 | 124823259 | NCOR2 | 0.934 | 0.919 | -0.015 | 3.62E-05 |  |
| cg21215436 | 9 | 137300911 | RXRA | 0.792 | 0.778 | -0.015 | 8.51E-04 |  |
| cg01283141 | 16 | 30020506 | DOC2A | 0.855 | 0.840 | -0.015 | 2.67E-05 |  |
| cg13856573 | 11 | 62475078 | BSCL2;GNG3 | 0.908 | 0.893 | -0.015 | 8.20E-05 |  |
| cg27483317 | 10 | 131517424 | MGMT | 0.749 | 0.734 | -0.015 | 7.08E-04 |  |
| cg04157979 | 13 | 113645505 | MCF2L | 0.878 | 0.864 | -0.015 | 3.95E-04 |  |
| cg22948319 | 19 | 39620117 | PAK4 | 0.935 | 0.921 | -0.014 | 1.68E-04 |  |
| cg19200561 | 6 | 116155750 |  | 0.866 | 0.851 | -0.014 | 4.83E-04 |  |
| cg23931121 | 1 | 27878094 | AHDC1 | 0.836 | 0.822 | -0.014 | 8.51E-04 |  |
| cg17330278 | 10 | 631304 | DIP2C | 0.897 | 0.883 | -0.014 | 5.86E-04 |  |
| cg17465569 | 1 | 22963605 | C1QA | 0.734 | 0.720 | -0.014 | 3.95E-04 |  |
| cg01603569 | 12 | 59925323 |  | 0.770 | 0.756 | -0.014 | 3.95E-04 |  |
| cg21635265 | 5 | 177035519 | B4GALT7 | 0.905 | 0.891 | -0.014 | 7.08E-04 |  |
| cg12157156 | 12 | 124880173 | NCOR2 | 0.938 | 0.924 | -0.014 | 4.77E-05 |  |
| cg18400182 | 17 | 80275724 | CD7 | 0.900 | 0.887 | -0.014 | 1.68E-04 |  |
| cg26846409 | 8 | 21640304 | GFRA2 | 0.600 | 0.586 | -0.014 | 5.86E-04 |  |
| cg13721800 | 19 | 7598343 | PNPLA6;MCOLN1 | 0.877 | 0.863 | -0.014 | 2.10E-04 |  |
| cg07824649 | 16 | 88569490 | ZFPM1 | 0.839 | 0.825 | -0.014 | 8.51E-04 |  |
| cg07478111 | 4 | 11401665 | HS3ST1 | 0.929 | 0.915 | -0.014 | 8.51E-04 |  |
| cg06718276 | 12 | 54134485 |  | 0.759 | 0.746 | -0.013 | 2.67E-05 |  |
| cg13027595 | 6 | 33283184 | ZBTB22;TAPBP | 0.792 | 0.778 | -0.013 | 4.77E-05 |  |
| cg05354904 | 16 | 88994375 | CBFA2T3 | 0.867 | 0.854 | -0.013 | 8.51E-04 |  |
| cg10061492 | 14 | 105849775 | PACS2 | 0.908 | 0.895 | -0.013 | 4.83E-04 |  |
| cg07988713 | 16 | 28388196 |  | 0.831 | 0.817 | -0.013 | 7.08E-04 |  |
| cg14329644 | 11 | 1253904 | MUC5B | 0.934 | 0.921 | -0.013 | 1.68E-04 |  |
| cg08959218 | 2 | 242887391 |  | 0.900 | 0.887 | -0.013 | 1.05E-04 |  |
| cg01652927 | 15 | 74229811 | LOXL1 | 0.919 | 0.906 | -0.013 | 5.86E-04 |  |
| cg09289463 | 19 | 5787866 | DUS3L | 0.906 | 0.893 | -0.013 | 4.83E-04 |  |
| cg04543077 | 16 | 2879544 | ZG16B | 0.838 | 0.825 | -0.013 | 8.51E-04 |  |
| cg13367644 | 8 | 1444085 |  | 0.937 | 0.924 | -0.013 | 3.22E-04 |  |
| cg24632843 | 1 | 179557954 |  | 0.932 | 0.919 | -0.013 | 8.51E-04 |  |
| cg19366986 | 1 | 3381321 | ARHGEF16 | 0.928 | 0.915 | -0.013 | 4.83E-04 |  |
| cg02482690 | 22 | 37449184 | KCTD17 | 0.914 | 0.901 | -0.013 | 7.08E-04 |  |
| cg00009796 | 16 | 33318910 |  | 0.780 | 0.767 | -0.013 | 4.77E-05 |  |
| cg16999584 | 6 | 169559716 |  | 0.867 | 0.854 | -0.013 | 3.62E-05 |  |
| cg23393892 | 7 | 2184028 | MAD1L1 | 0.908 | 0.896 | -0.013 | 2.67E-05 |  |
| cg17028218 | 11 | 67261122 | PITPNM1 | 0.838 | 0.826 | -0.013 | 3.22E-04 |  |
| cg03139255 | 6 | 33383093 | PHF1 | 0.860 | 0.847 | -0.013 | 8.51E-04 |  |
| cg09333109 | 19 | 51532580 | KLK11;KLK12 | 0.935 | 0.922 | -0.013 | 3.22E-04 |  |
| cg07803733 | 6 | 169648038 | THBS2 | 0.754 | 0.742 | -0.012 | 2.61E-04 |  |
| cg10502563 | 6 | 32134694 | EGFL8 | 0.817 | 0.805 | -0.012 | 4.83E-04 |  |
| cg03038418 | 19 | 3096512 | GNA11 | 0.674 | 0.662 | -0.012 | 7.08E-04 |  |
| ch.17.72493169F | 17 | 74981574 |  | 0.154 | 0.142 | -0.012 | 5.86E-04 |  |
| cg02556381 | 1 | 247463421 |  | 0.925 | 0.913 | -0.012 | 7.08E-04 |  |
| cg00628375 | 17 | 42390584 | RUNDC3A | 0.919 | 0.907 | -0.012 | 1.68E-04 |  |
| cg05575304 | 3 | 11607115 | VGLL4 | 0.931 | 0.919 | -0.012 | 1.34E-05 |  |
| cg17241447 | 19 | 8201349 | FBN3 | 0.903 | 0.891 | -0.012 | 1.34E-05 |  |
| cg22054580 | 8 | 144811385 | FAM83H | 0.874 | 0.862 | -0.012 | 1.34E-04 |  |
| cg21289763 | 17 | 78868835 | RPTOR | 0.834 | 0.822 | -0.012 | 8.51E-04 |  |
| cg17018896 | 7 | 1940054 | MAD1L1 | 0.856 | 0.844 | -0.012 | 7.08E-04 |  |
| cg10951325 | 6 | 31838419 | SLC44A4 | 0.841 | 0.829 | -0.012 | 4.83E-04 |  |
| cg02138198 | 12 | 57485116 | NAB2 | 0.863 | 0.851 | -0.012 | 7.08E-04 |  |
| cg16944597 | 3 | 184055379 | FAM131A | 0.864 | 0.852 | -0.012 | 8.51E-04 |  |
| cg25310555 | 13 | 110434682 | IRS2 | 0.927 | 0.916 | -0.012 | 3.22E-04 |  |
| cg26062856 | 15 | 26109425 | ATP10A | 0.690 | 0.678 | -0.012 | 1.34E-05 |  |
| cg00885023 | 17 | 25309298 |  | 0.946 | 0.935 | -0.012 | 2.61E-04 |  |
| cg13897316 | 5 | 32582081 |  | 0.929 | 0.918 | -0.012 | 4.83E-04 |  |
| cg14021321 | 9 | 137594239 | COL5A1 | 0.931 | 0.920 | -0.012 | 2.61E-04 |  |
| cg02022978 | 14 | 40817444 |  | 0.885 | 0.874 | -0.012 | 8.51E-04 |  |
| cg01816880 | 1 | 228404896 | OBSCN | 0.829 | 0.817 | -0.011 | 3.95E-04 |  |
| cg15086199 | 6 | 33362999 | KIFC1 | 0.863 | 0.851 | -0.011 | 3.95E-04 |  |
| cg20699151 | 5 | 8840460 |  | 0.708 | 0.696 | -0.011 | 3.62E-05 |  |
| cg05766129 | 3 | 123988013 | KALRN | 0.912 | 0.901 | -0.011 | 3.81E-06 |  |
| cg23817643 | 10 | 134164721 | LRRC27 | 0.922 | 0.910 | -0.011 | 8.51E-04 |  |
| cg12899248 | 17 | 15845357 |  | 0.931 | 0.920 | -0.011 | 5.86E-04 |  |
| cg12969734 | 22 | 50962224 | SCO2 | 0.925 | 0.914 | -0.011 | 2.61E-04 |  |
| cg18394881 | 1 | 3512777 | MEGF6 | 0.943 | 0.932 | -0.011 | 4.83E-04 |  |
| cg20194872 | 6 | 41519635 | FOXP4 | 0.920 | 0.909 | -0.011 | 2.10E-04 |  |
| cg09326702 | 11 | 64323448 | SLC22A11 | 0.931 | 0.920 | -0.011 | 3.95E-04 |  |
| cg20118823 | 19 | 1612208 | TCF3 | 0.925 | 0.914 | -0.011 | 2.10E-04 |  |
| cg00109293 | 1 | 3149830 | PRDM16 | 0.835 | 0.824 | -0.011 | 3.22E-04 |  |
| cg15677989 | 1 | 32208487 | BAI2 | 0.839 | 0.828 | -0.011 | 5.86E-04 |  |
| cg17320856 | 12 | 132687617 | GALNT9 | 0.853 | 0.843 | -0.011 | 8.51E-04 |  |
| cg22381282 | 5 | 180219882 | MGAT1 | 0.897 | 0.887 | -0.011 | 4.83E-04 |  |
| cg22402261 | 1 | 36026611 | NCDN | 0.912 | 0.902 | -0.011 | 4.83E-04 |  |
| cg10418812 | 7 | 1850329 |  | 0.935 | 0.925 | -0.010 | 5.86E-04 |  |
| cg22744555 | 4 | 2306425 | ZFYVE28 | 0.923 | 0.912 | -0.010 | 5.86E-04 |  |
| cg23693365 | 17 | 19374174 |  | 0.911 | 0.900 | -0.010 | 2.10E-04 |  |
| cg19768125 | 1 | 27917983 | AHDC1 | 0.939 | 0.929 | -0.010 | 3.95E-04 |  |
| cg25372239 | 1 | 3130550 | PRDM16 | 0.928 | 0.918 | -0.010 | 3.95E-04 |  |
| cg09934852 | 19 | 14045685 | PODNL1 | 0.789 | 0.779 | -0.010 | 3.22E-04 |  |
| cg16059943 | 1 | 2013748 | PRKCZ | 0.953 | 0.943 | -0.010 | 8.51E-04 |  |
| cg18013426 | 17 | 3512886 | SHPK;TRPV1 | 0.914 | 0.904 | -0.010 | 4.83E-04 |  |
| cg27232130 | 6 | 163607984 | PACRG | 0.795 | 0.785 | -0.010 | 1.34E-04 |  |
| cg08731067 | 11 | 3164631 | OSBPL5 | 0.940 | 0.931 | -0.010 | 5.86E-04 |  |
| cg16664496 | 16 | 87740413 | LOC100129637 | 0.852 | 0.842 | -0.010 | 1.34E-04 |  |
| cg26093898 | 17 | 80137377 | CCDC57 | 0.946 | 0.936 | -0.010 | 3.62E-05 |  |
| cg21982924 | 6 | 31516059 | ATP6V1G2;NFKBIL1 | 0.854 | 0.844 | -0.010 | 7.08E-04 |  |
| cg03661054 | 2 | 3281330 | TSSC1 | 0.919 | 0.909 | -0.010 | 8.20E-05 |  |
| cg09132923 | 17 | 8108163 | AURKB | 0.877 | 0.868 | -0.010 | 5.86E-04 |  |
| cg05256656 | 16 | 67290583 | SLC9A5 | 0.804 | 0.795 | -0.010 | 8.51E-04 |  |
| cg13119928 | 12 | 53228661 | KRT79 | 0.878 | 0.869 | -0.009 | 1.34E-04 |  |
| cg11926510 | 1 | 4832380 | AJAP1 | 0.952 | 0.942 | -0.009 | 5.86E-04 |  |
| cg05623781 | 13 | 114807633 | RASA3 | 0.945 | 0.936 | -0.009 | 2.10E-04 |  |
| cg06673083 | 19 | 2344540 | SPPL2B | 0.842 | 0.832 | -0.009 | 6.29E-05 |  |
| cg16016036 | 2 | 1417109 | TPO | 0.927 | 0.918 | -0.009 | 6.29E-05 |  |
| cg11558576 | 8 | 63056186 |  | 0.900 | 0.891 | -0.009 | 3.22E-04 |  |
| cg05142023 | 16 | 86570343 | MTHFSD | 0.905 | 0.896 | -0.009 | 8.51E-04 |  |
| cg16491103 | 8 | 54573876 |  | 0.934 | 0.926 | -0.009 | 8.51E-04 |  |
| cg01950810 | 6 | 31598385 | BAT2 | 0.937 | 0.928 | -0.009 | 5.86E-04 |  |
| ch.11.1613249R | 11 | 74547086 | RNF169 | 0.052 | 0.043 | -0.009 | 4.83E-04 |  |
| cg18214436 | 12 | 120565257 | GCN1L1 | 0.873 | 0.864 | -0.009 | 7.08E-04 |  |
| cg22329201 | 4 | 3569189 |  | 0.777 | 0.769 | -0.009 | 3.95E-04 |  |
| cg05251593 | 17 | 55517831 | MSI2 | 0.773 | 0.765 | -0.009 | 5.86E-04 |  |
| cg03754708 | 10 | 134604989 |  | 0.626 | 0.617 | -0.009 | 1.68E-04 |  |
| cg10256255 | 2 | 174890794 |  | 0.933 | 0.925 | -0.008 | 7.08E-04 |  |
| cg19324627 | 11 | 116708329 | APOA1 | 0.928 | 0.920 | -0.008 | 7.08E-04 |  |
| cg04549287 | 13 | 52952319 | THSD1 | 0.881 | 0.873 | -0.008 | 4.83E-04 |  |
| cg13554549 | 22 | 47191940 | TBC1D22A | 0.831 | 0.823 | -0.008 | 3.22E-04 |  |
| cg01669732 | 19 | 14074320 | RFX1 | 0.920 | 0.912 | -0.008 | 4.83E-04 |  |
| cg11538410 | 8 | 142141455 | DENND3 | 0.944 | 0.936 | -0.008 | 4.83E-04 |  |
| cg05364651 | 16 | 1267876 | CACNA1H | 0.905 | 0.897 | -0.008 | 8.51E-04 |  |
| ch.2.171328329F | 2 | 171620083 |  | 0.065 | 0.057 | -0.008 | 1.34E-04 |  |
| cg01056398 | 3 | 53857698 | CHDH | 0.935 | 0.927 | -0.008 | 8.51E-04 |  |
| ch.4.188624547F | 4 | 188387553 |  | 0.074 | 0.066 | -0.008 | 4.83E-04 |  |
| cg19230443 | 19 | 49950584 | PIH1D1 | 0.932 | 0.924 | -0.008 | 4.83E-04 |  |
| cg23792115 | 17 | 79254229 | SLC38A10 | 0.920 | 0.912 | -0.008 | 1.34E-05 |  |
| cg24687253 | 7 | 100865887 | ZNHIT1 | 0.952 | 0.944 | -0.008 | 7.08E-04 |  |
| cg09779544 | 11 | 133777981 |  | 0.950 | 0.943 | -0.008 | 5.86E-04 |  |
| cg07558837 | 6 | 160555312 | SLC22A1 | 0.949 | 0.941 | -0.008 | 5.86E-04 |  |
| cg06309629 | 10 | 4076396 |  | 0.926 | 0.918 | -0.008 | 5.86E-04 |  |
| cg16145428 | 2 | 196412368 |  | 0.924 | 0.917 | -0.008 | 8.51E-04 |  |
| cg23349798 | 6 | 148841330 | SASH1 | 0.934 | 0.926 | -0.008 | 7.08E-04 |  |
| ch.X.92554290F | X | 92667634 |  | 0.047 | 0.040 | -0.008 | 1.34E-04 |  |
| cg07305000 | 7 | 157602227 | PTPRN2 | 0.955 | 0.948 | -0.007 | 3.95E-04 |  |
| cg09910120 | 10 | 131190389 |  | 0.953 | 0.946 | -0.007 | 4.83E-04 |  |
| cg23919549 | 19 | 576047 | BSG | 0.957 | 0.950 | -0.007 | 3.95E-04 |  |
| cg06909646 | 1 | 32681593 | DCDC2B | 0.871 | 0.863 | -0.007 | 8.51E-04 |  |
| cg03711562 | 17 | 18936298 | GRAP | 0.906 | 0.899 | -0.007 | 7.08E-04 |  |
| cg25634017 | 7 | 16438022 | ISPD | 0.958 | 0.951 | -0.007 | 4.83E-04 |  |
| cg27055366 | 14 | 105916228 | MTA1 | 0.897 | 0.890 | -0.007 | 5.86E-04 |  |
| cg05625074 | 4 | 679907 | MFSD7 | 0.879 | 0.872 | -0.007 | 1.68E-04 |  |
| cg05197164 | 13 | 113724548 | MCF2L | 0.932 | 0.925 | -0.007 | 2.67E-05 |  |
| cg22168222 | 7 | 2584609 | C7orf27 | 0.930 | 0.923 | -0.007 | 4.83E-04 |  |
| cg03075156 | 2 | 45986586 | PRKCE | 0.954 | 0.947 | -0.007 | 7.08E-04 |  |
| cg06900311 | 14 | 106333919 |  | 0.954 | 0.946 | -0.007 | 1.34E-04 |  |
| cg24393700 | 12 | 133008035 |  | 0.951 | 0.944 | -0.007 | 6.29E-05 |  |
| cg07269819 | 20 | 62196641 | PRIC285 | 0.962 | 0.955 | -0.007 | 3.95E-04 |  |
| cg06903451 | 1 | 1323192 | CCNL2 | 0.929 | 0.922 | -0.007 | 2.61E-04 |  |
| cg26695117 | 17 | 64264320 |  | 0.949 | 0.942 | -0.007 | 1.68E-04 |  |
| cg02927694 | 22 | 38077057 |  | 0.841 | 0.834 | -0.007 | 4.83E-04 |  |
| cg17278890 | 10 | 5541726 | CALML5 | 0.949 | 0.942 | -0.007 | 5.86E-04 |  |
| cg11773367 | 11 | 85484509 | SYTL2 | 0.944 | 0.938 | -0.007 | 7.08E-04 |  |
| cg22467232 | 5 | 1816093 | NDUFS6 | 0.933 | 0.927 | -0.007 | 1.68E-04 |  |
| cg24659171 | 11 | 67817691 | TCIRG1 | 0.898 | 0.892 | -0.007 | 4.77E-05 |  |
| cg26221342 | 5 | 1272400 | TERT | 0.943 | 0.937 | -0.006 | 5.86E-04 |  |
| cg06976482 | 10 | 134913812 | GPR123 | 0.924 | 0.918 | -0.006 | 8.51E-04 |  |
| cg14312783 | 10 | 131504572 | MGMT | 0.908 | 0.901 | -0.006 | 8.51E-04 |  |
| cg23645149 | 17 | 35870381 | DUSP14 | 0.935 | 0.928 | -0.006 | 7.08E-04 |  |
| cg01167623 | 16 | 21719790 | OTOA | 0.952 | 0.946 | -0.006 | 7.08E-04 |  |
| cg04021706 | 5 | 389763 | AHRR | 0.952 | 0.946 | -0.006 | 5.86E-04 |  |
| cg15869071 | 4 | 2798158 | SH3BP2 | 0.953 | 0.947 | -0.006 | 7.08E-04 |  |
| ch.2.154234353F | 2 | 154526107 |  | 0.049 | 0.043 | -0.006 | 4.77E-05 |  |
| cg17220512 | 21 | 15053472 |  | 0.950 | 0.944 | -0.006 | 8.51E-04 |  |
| cg08796458 | 22 | 46640902 | C22orf40 | 0.942 | 0.936 | -0.006 | 7.08E-04 |  |
| cg27110655 | 19 | 48259098 | GLTSCR2;SNORD23 | 0.870 | 0.864 | -0.006 | 4.83E-04 |  |
| cg02433807 | 16 | 11234924 | CLEC16A | 0.930 | 0.924 | -0.006 | 4.83E-04 |  |
| cg07699277 | 6 | 34004226 | GRM4 | 0.960 | 0.954 | -0.006 | 3.95E-04 |  |
| cg26406558 | 13 | 99174395 | STK24 | 0.846 | 0.840 | -0.006 | 5.86E-04 |  |
| cg19169838 | X | 153631347 | DNASE1L1 | 0.957 | 0.951 | -0.005 | 7.08E-04 |  |
| cg12065406 | 1 | 180165648 | QSOX1;FLJ23867 | 0.964 | 0.959 | -0.005 | 8.51E-04 |  |
| cg24502933 | 1 | 170136883 | METTL11B | 0.951 | 0.945 | -0.005 | 8.20E-05 |  |
| cg18039933 | 12 | 133132641 | FBRSL1 | 0.947 | 0.942 | -0.005 | 5.86E-04 |  |
| cg03193893 | 6 | 30644478 | KIAA1949 | 0.942 | 0.936 | -0.005 | 4.83E-04 |  |
| cg21408848 | 3 | 12949849 | IQSEC1 | 0.953 | 0.948 | -0.005 | 5.86E-04 |  |
| cg01128412 | 2 | 242787064 |  | 0.958 | 0.953 | -0.005 | 5.86E-04 |  |
| ch.10.181390F | 10 | 5940801 | FBXO18 | 0.040 | 0.035 | -0.005 | 7.08E-04 |  |
| ch.15.77890571R | 15 | 80103516 |  | 0.032 | 0.027 | -0.005 | 5.86E-04 |  |
| ch.20.12629947R | 20 | 12681947 |  | 0.034 | 0.030 | -0.005 | 4.83E-04 |  |
| cg00236249 | 22 | 20789017 | SCARF2 | 0.941 | 0.936 | -0.005 | 3.95E-04 |  |
| cg00396981 | 16 | 57787375 | KATNB1 | 0.979 | 0.975 | -0.004 | 4.83E-04 |  |
| cg23795429 | 7 | 73152092 | ABHD11 | 0.953 | 0.948 | -0.004 | 3.62E-05 |  |
| cg00551487 | 7 | 151091744 | WDR86 | 0.864 | 0.860 | -0.004 | 5.86E-04 |  |
| cg01400924 | 12 | 124947742 | NCOR2 | 0.917 | 0.913 | -0.004 | 7.08E-04 |  |
| cg23603789 | 2 | 242572545 | THAP4 | 0.951 | 0.947 | -0.004 | 2.10E-04 |  |
| ch.10.25135601F | 10 | 25095597 |  | 0.039 | 0.036 | -0.003 | 8.51E-04 |  |
| cg01979352 | 1 | 182569418 | RGS16 | 0.673 | 0.670 | -0.003 | 7.08E-04 |  |
| cg07217563 | 10 | 1149628 | WDR37 | 0.968 | 0.964 | -0.003 | 2.10E-04 |  |
| cg26849909 | 17 | 79177197 | AZI1 | 0.932 | 0.930 | -0.003 | 5.86E-04 |  |
| cg21384883 | X | 48457289 | WDR13 | 0.952 | 0.955 | 0.003 | 3.22E-04 |  |
| cg19968916 | 10 | 135087441 | ADAM8 | 0.970 | 0.973 | 0.003 | 2.67E-05 |  |
| cg00110609 | 16 | 4311799 | TFAP4 | 0.951 | 0.956 | 0.005 | 7.08E-04 |  |
| cg26910936 | 5 | 173661962 |  | 0.941 | 0.949 | 0.007 | 2.10E-04 |  |
| cg00874558 | 8 | 2021720 | MYOM2 | 0.905 | 0.914 | 0.009 | 2.10E-04 |  |
| cg21162356 | 1 | 226668457 |  | 0.931 | 0.942 | 0.011 | 3.95E-04 |  |
| cg16183122 | 10 | 88466389 | LDB3 | 0.915 | 0.926 | 0.012 | 4.83E-04 |  |
| cg04145643 | 1 | 45288012 | PTCH2 | 0.890 | 0.902 | 0.012 | 1.34E-04 |  |
| cg01561807 | 13 | 114307602 | ATP4B | 0.884 | 0.896 | 0.012 | 1.34E-04 |  |
| cg19044706 | 2 | 241829685 | C2orf54 | 0.928 | 0.940 | 0.013 | 2.61E-04 |  |
| cg15956469 | 12 | 10465272 | KLRD1 | 0.891 | 0.905 | 0.014 | 2.10E-04 |  |
| cg05680710 | 6 | 31868144 | ZBTB12 | 0.876 | 0.891 | 0.015 | 8.51E-04 |  |
| cg01647795 | 15 | 44969244 | PATL2 | 0.898 | 0.917 | 0.019 | 5.86E-04 |  |
| cg26581228 | 6 | 42326264 | TRERF1 | 0.904 | 0.924 | 0.020 | 1.68E-04 |  |
| cg00871381 | 19 | 3879827 | ATCAY | 0.809 | 0.836 | 0.027 | 1.68E-04 |  |
| cg01157780 | 20 | 34700376 | EPB41L1 | 0.604 | 0.635 | 0.031 | 1.05E-04 |  |
| cg06935361 | 13 | 32964454 | BRCA2 | 0.678 | 0.727 | 0.049 | 7.08E-04 |  |
| cg12803068 | 7 | 45002919 | MYO1G | 0.758 | 0.842 | 0.083 | 3.95E-04 | |
